# Effect of dietary *Bacillus subtilis*, dried whey powder, and *Saccharomyces cerevisiae* on the production performance, egg quality, blood biochemical parameters, and ileal histomorphology in late-phase Lohmann LSL- lite laying hens

**DOI:** 10.1016/j.psj.2025.105547

**Published:** 2025-07-07

**Authors:** Zahra Hamzehee, Mehran Torki, Khodabakhsh Rashidi, Alireza Abdolmohammadi

**Affiliations:** a Department of Animal Science, College of Agriculture and Natural Resources, Razi University, Kermanshah, Iran; b Research Center of Oils and Fats, Kermanshah University of Medical Sciences, Kermanshah, Iran

**Keywords:** *Bacillus subtilis*, Prebiotic, Probiotic, *Saccharomyces cerevisia*e, Whey powder

## Abstract

The current study was conducted to investigate the effects of dietary whey powder (WP), *Bacillus subtilis* (*B. subtilis*), and *Saccharomyces cerevisiae* (*S. cerevisiae*) and their combination on the production performance, egg quality, blood biochemical and ileum histomorphology parameters of Lohmann laying hens. A total of 288 Lohmann LSL-Lite laying hens (75 wk) were randomly allocated to 8 treatments with 6 replicates of 6 hens each, over a 12-week period. A 2 × 2 × 2 factorial arrangement was used, consisting of a basal corn-soybean meal diet with two levels of *B. subtilis* (0 and 1 g/kg diet, 10^9^ CFU), two levels of WP (0 and 1 g/kg diet), and two levels of *S. cerevisiae* (0 and 3 g/kg diet, 5 × 10^9^ CFU). The control group received a standard diet without any additive. No significant three-way interaction among WP, *B. subtilis*, and *S. cerevisiae* was observed for the studied parameters, except for malondialdehyde level (*P* < 0.05), where a significant three-way interaction was detected, resulting in reduced malondialdehyde concentrations (*P* = 0.028). The simultaneous inclusion of WP and *B. subtilis* improved ileum villus length and serum calcium levels. A significant two-way interaction between WP and *B. subtilis* indicated a synergistic effect on egg production, weight, and mass during 75–80 weeks (*P* < 0.05). In conclusion, it seems that the simultaneous use of WP and *B. subtilis* is effective in improving performance and ileum histomorphology in Lohmann laying hens in the late production phase.

## Introduction

The laying period from 45 to 72 weeks of age is known as the late phase of hen laying. Aging is a natural and irreversible physiological process that is associated with progressive mitochondrial dysfunction and cumulative metabolic stress, leading to increased production of harmful reactive oxygen species (ROS) (Lee et al., 2004). As hens age, the balance between ROS generation and antioxidant defense systems is disrupted, largely due to a gradual decline in the activity of endogenous antioxidant enzymes such as superoxide dismutase and glutathione peroxidase (Tong et al., 2015). This imbalance results in oxidative stress, which negatively impacts cellular function, reproductive hormone secretion, and intestinal health, ultimately contributing to reduced production performance and egg quality in late-phase laying (Park and Sohn, 2018; Yu et al., 2023). Egg production and quality, including eggshell strength and albumen height, decline swiftly at the end of the laying cycle (Liu et al., 2018). Thus, strategies are needed to enhance egg production and quality during the late laying period, thereby extending the laying cycle and improving breeding efficiency in hens (Guo et al., 2020; Zhang et al., 2019).

Probiotics, also known as direct-fed microbials, are live non-pathogenic microorganisms that, when administered in the right quantity, provide significant health and performance benefits to the host by enhancing microbial balance (FAO/WHO, 2002). Proposed mechanisms include the secretion of antimicrobial substances, competitive adherence to the mucosa and epithelium, the strengthening of the gut epithelial barrier, and the modulation of the immune system (Bai et al., 2013; Broom and Kogut, 2018). *B. subtilis*, a resilient spore-forming probiotic, can endure various environmental challenges, including high temperatures and extreme pH levels (Cai et al., 2024; Nicholson et al., 2012). *Bacillus* spores exhibit remarkable survival capabilities in harsh gastrointestinal circumstances, including exposure to bile salts and low pH (Shivaramaiah et al., 2011). Studies indicate that incorporating *B. subtilis* (300 and 500 mg/kg) into broiler diets reduces pathogen growth, alters gut flora, decreases intestinal inflammation, and modifies mucosal structure, all of which enhance poultry production (Cai et al., 2024; Park et al., 2020; Wang et al., 2021).

The gut microbiota, a crucial component of the host organism, exerts significant influence on the bioavailability of dietary components and plays a pivotal role in the host’s nutritional and physiological processes. Research suggests that the ability of probiotics to balance intestinal microflora may contribute to enhanced animal growth performance and improved intestinal function (Ma et al., 2018). Recent research has shown that *B. subtilis* can inhibit *Salmonella* growth directly and may also enhance short-chain fatty acids (SCFAs), particularly acetate, propionate, and butyrate, by improving intestinal microbiota composition (Neijat et al., 2019). SCFAs are known to boost host nonspecific immunity and directly suppress *Salmonella* (Tsugawa et al., 2020). *B. subtilis* demonstrated effective protection against *Salmonella pullorum* in chickens, evidenced by reduced mortality, less inflammatory injury, and increased expression of tight junction genes (Wang et al., 2022). Recent meta-analyses have highlighted the significant benefits of supplementing poultry diets with *B. subtilis* and prebiotics. Ningsih et al. (2023) demonstrated that dietary inclusion of *B. subtilis* as a probiotic in broiler chickens with *Necrotic enteritis* not only improved final body weight and average daily gain but also produced comparable results to antibiotic treatments, underscoring its potential as a natural growth promoter and health enhancer in poultry production.

Moreover, dietary *B. subtilis* supplementation increased the population of immune response-related cells in the intestinal mucosa and number of individual lymphoid follicles occurring in the lamina propria of the mucosa in chickens receiving *B. subtilis* (Molnár et al., 2011). Administering a diet supplemented with microencapsulated *Enterococcus faecium, Lactobacillus plantarum*, and *B. subtilis* positively affects the levels of immunoglobulins M and A, and it also increases the percentage of total antioxidant capacity in serum (Wang et al., 2018). Research has shown that Supplemental *B. subtilis* increased average body weight and average daily gain, as well as elevated villus height and villus height to crypt depth ratio of ileum in broilers (Ma et al., 2018).

The fresh liquid from cheese making contains 93.4 % water, 0.76 % whey protein, 0.61 % minerals, 0.09 % fat, and 5.12 % lactose, which is its main ingredient (USDA, 2018). Lactose is not digested, and is thus fermented by the cecal microflora, which could decrease pH, promote lactic-acid bacteria growth, and suppress pathogenic bacteria (Abd El-Hack et al., 2022; Gülşen et al., 2002). Lactose is passed down to a lower bowel where beneficial bacteria will ferment it and produce SCFAs such as butyrate, propionate, and acetate (Pineda-Quiroga et al., 2017; Torres-Rodriguez et al., 2007). An increase in the concentration of SCFAs is often associated with an increase in the population of useful bacteria and a decrease in pathogenic microorganisms, which can lead to the stabilization of microbial eubiosis in the gastrointestinal tract (Shabani et al., 2019). The main components of whey proteins include α-lactalbumin, β-lactoglobulin, bovine serum albumin, immunoglobulins, bovine lactoferrin, bovine Lactoperoxidase, and minor amounts glycomacropeptide (Minj and Anand, 2020). Based on these properties, WP can be considered a feed additive with prebiotic-like effects in poultry feed.

Prebiotics are in-digestible or poorly digestible feed ingredients that provide benefits to the host through selectively stimulating bacterial growth and also activating the metabolism of health-promoting bacteria located in the gut, thereby improving the host's microbial balance (Gibson and Roberfroid, 1995). The yeast *S. cerevisiae* is oval, white, or cream, and reproduces vegetatively by budding and sexually by cystic spores (Barnett et al., 1990; Firman et al., 2013). *S. cerevisiae* produces nutrients such as amino acids and vitamins, and enzymes such as glucanases, mannanases, amylases, proteases, and lipases. The yeast cell wall matrix consists mostly of α-mannan (31 % dry mass); mannoproteins (mannan + protein), together accounting for about 40 % dry mass, β-glucan (about 60 % dry mass), and chitin (about 2 % dry mass) (Ahiwe et al., 2021). The dietary inclusion of *S. cerevisiae* significantly improved the FCR and egg production and reduced the feed intake in laying Japanese quail (Zamanizadeh et al., 2021). However, feed consumption, egg production per day, egg weight, and FCR were not affected by the use of live yeast (Yalçın et al., 2015). Several studies have found a significant increase in egg production (Berry and Lui, 2000; Yalçın et al., 2010) and FCR (Kumprecht et al., 1997; Yalçın et al., 2010) in chickens that were fed with yeast and yeast products. This improvement may be due in part to the enhancement of gut health and nutrient absorption (Yalçin et al., 2008). Furthermore, Adli et al. (2023) conducted a meta-regression analysis and reported that supplementation with oligosaccharides (a class of prebiotics) non-linearly increased hen-day egg production and improved antioxidant status, as evidenced by enhanced superoxide dismutase activity and reduced malondialdehyde concentrations. However, oligosaccharide supplementation did not significantly affect feed intake, feed conversion ratio, egg mass, egg weight, eggshell quality, or Haugh unit. These findings collectively suggest that both *B. subtilis* and prebiotics can play important roles in supporting gut health, immune function, and productive performance in laying hens, especially during the late laying period when birds are more susceptible to physiological stress and production decline.

*S. cerevisiae* is a single-celled Gram-positive yeast that cannot adhere to the intestinal wall, but it can consume high amounts of oxygen, creating anaerobic conditions that promote the growth and multiplication of lactobacilli (Barnett et al., 1990). *S. cerevisiae*, also known as baker's yeast, is a powerful source of glucan fiber that can effectively help to reduce cholesterol levels through various mechanisms. One of these mechanisms involves its binding with bile acids in the digestive tract, which results in a lower amount of bile acids being reabsorbed by the liver(Zahira and AL-Tabari, 2014). Ducks fed fermented *S. cerevisiae* improved almost 5.24 % and 4.26 % higher egg mass and hen-day egg production, respectively (Hossain et al., 2024). To the best of our knowledge, no previous studies have investigated the combined effects of WP, *B. subtilis*, and *S. cerevisiae* in late-phase laying hens. Given their complementary biological functions, WP as a source of bioactive peptides and prebiotics, *B. subtilis* as a probiotic enhancing gut microbiota and enzyme activity, and *S. cerevisiae* as a yeast probiotic improving intestinal health and immune response, we hypothesized that their individual and combined supplementation would improve production performance, egg quality, intestinal morphology, and blood biochemical parameters during the late laying phase. Furthermore, we aimed to determine whether their combined use could provide additive or synergistic benefits beyond those observed with individual supplementation.

## Materials and methods

### Birds and Diets

A total of 450 fertile eggs were purchased from a local hatchery and incubated in the Animal Science Department laboratory. Hatched chicks were reared according to the company catalogue until the start of the experimental trial. Before the start of the experiment, all hens were individually weighed, and their egg production rate and egg weight were recorded for two consecutive weeks. Based on these records, 288 laying hens with similar body weight, egg production, and egg weight were selected and then randomly assigned to 48 conventional cages (*n* = 6 per cage) in such a way that the initial means and variances for these parameters were similar across all groups. Thus, the hens used in this study were not privately owned by another institution, individual, or farm.

A total of two hundred and eighty-eight 75-week-old laying hens (LSL-Lite) were randomly divided into 8 groups with six replicates of six birds each (3 birds per conventional cage, 44 × 40 × 40 cm; no enrichment provisions) according to a completely randomized design, for 12 wk. The hens were provided with water through nipple drinkers, which ensured hygienic and continuous access to fresh water throughout the experimental period. The experimental design was a factorial arrangement evaluating the presence or absence of three dietary additives: 1 g/kg *B. subtilis* (powdered form, 10^9^ CFU/g, Parsilact Co., Shiraz, Iran), 1 g/kg WP, and 3 g/kg *S. cerevisiae* (granulated form, 5 × 10^9^ supplied by a Fartak Co, Iran). The experimental diets consisted of a basal corn-soybean meal or control, control diet + 1 g/kg *B. subtilis*, control diet + 1 g/kg WP, control diet + 3 g/kg *S. cerevisiae*, control diet + 1 g/kg WP and *B. subtilis*, control diet + 3 g/kg *S. cerevisiae,* and1 g/kg *B. subtilis*, control diet + 1 g/kg WP and 3 g/kg *S. cerevisiae* and combination of all three *S. cerevisiae* (3 g/kg), WP (1 g/kg), *B. subtilis* (1 g/kg). The hens were housed under controlled environmental conditions with a lighting schedule of 16 hours light and 8 hours dark. Ventilation was maintained to ensure adequate air exchange, and relative humidity was controlled between 60 and 70 % throughout the experimental period.

### Management and sample collection

During the experiment, the hens were provided with feed and water ad libitum, and the hen-house temperature was kept between 20 and 22°C. The basal diets are shown in Table 1. Hens were weighed individually at the beginning and the end of the experiment. Feed was supplied to the birds two times per day, and FI was calculated by weighing the remaining feed at the end of each week. FCR was calculated as kilograms of feed per kilogram of egg. All of the eggs were collected and weighed individually to determine the egg weight. Using these values, egg production, egg weight, and daily egg yield were calculated. Egg quality was determined at the end of the trial. Six eggs were collected randomly from each replication in the experiment. Shell thickness was measured in 3 different parts (upper and lower ends and middle) by a micrometer (Baxlo, 0.01 mm). Yolk color was determined according to the Roche Yolk color fan. Haugh unit was determined by measuring the height of the thick albumen using a Haugh Tester (Baxio, Spain) and the weight of the egg using a digital scale. The Haugh unit was then calculated using the following formula:

(1) $$\text{Haugh}\text{units}\left(\%\right):100\times \text{log}\left(H+7.57-1.7W^{0.37}\right)$$

where H is the height of the albumen and W is the weight of the egg.

**Table 1:** Composition and nutrient contents of basal diet fed in the experimental diet of laying hens.

Ingredients (%)	amount	calculate	amount
Corn	58.32	ME (Kcal/kg)	2700
Barley	2.00	CP (%)	15.4
Soybean meal	24.00	Lys (%)	0.70
Wheat bran	1.84	Met (%)	0.40
Nacl	0.18	Met + Cys (%)	0.63
Vit & min Premix ^1^	0.60	Thr (%)	0.49
DL-Methionine	0.20	AP (%)	0.30
bicarbonate sodium	0.24		
DCP	1.24		
Limestone	9.61		
toxin Binder	0.10		

1. Vitamin and mineral premix provided (units per kg/feed): VA 9000 IU, VD3 2000 IU, VE 20 IU, VK 3 mg, VB1 1 mg, VB2 6 mg, VB6 5 mg, VB12 20 mcg, calcium pantothenate 30 mg, nicotinic acid 26 mg, biotin 38 mcg, folic acid 0.4 mg, choline 400 mg, iron, 25 mg; iodine, 0.45 mg; manganese, 80 mg; zinc, 60 mg; selenium, 0.2 mg.100.000 mg, Cobalt: 200 mg, Copper: 4.000 mg, Zinc Bacitracin: 21.000 mg.

Specific gravity was determined using the method recommended by (Butcher and Miles, 1991).

At the end of the experiment, one hen per replicate was randomly selected for blood sampling by assigning a number to each bird and using a random number generator. Blood samples were collected from the brachial vein, allowed to solidify, centrifuged (3000 rpm for 15 min at 4°C), and the serum was extracted. Serum samples were taken and stored under (−20 °C) until assayed for estimation of Serum Glucose, cholesterol, triglyceride, total protein, albumin, phosphorus, calcium, uric acid, malondialdehyde, and total antioxidant capacity using standard kits (Pars Azmoon Co., Tehran, Iran; Delta Darman Part, Tehran, Iran).

### Ileum histomorphology characteristics

At the end of the experimental period, one randomly selected bird per replicate was anaesthetized via inhalation of 60 % CO2 and then sacrificed by cervical dislocation. No bird died before meeting the criteria for euthanasia. A sample (2 cm in length) was taken from the end of the ileum. After rinsing with distilled water, it was moved to the sample container containing 10 % formaldehyde. The tissue processing machine (Dideh Sabz Co. Model D2080/H, manufactured in Iran) was used to prepare the microscopic sections. The tissue samples were embedded in histological paraffin, sliced into sections using a rotary microtome (Leica), and stained with hematoxylin and eosin. All samples were examined after preparation. The villi were evaluated based on the height, width, crypt depth, and villus height to crypt depth ratio.

### Data analysis

At first, data normality was verified using the Shapiro-Wilk normality test. Data were compared using the General Linear Model (GLM) procedure followed by Duncan’s multiple range test (*P* < 0.05) with SAS 9.4 software and level of 0.05 was considered to show significant differences among means. In this experiment, 2 × 2 × 2 factorial arrangements were used. To perform variance analysis, the following statistical model was considered (Equation (2)):

(2) $$Y_{ijk}=\;\mu \;+A_{i}\;+\;B_{j}\;+\;C_{k}\;+\;{\left(AB\right)}_{ij}\;+\;{\left(AC\right)}_{ik}\;+\;{\left(BC\right)}_{jk}\;+\;{\left(ABC\right)}_{ijk}\;+\;e_{ijk}$$

Where $Y_{ijk}$ is the observed response, μ is the overall mean, $A_{i}$, $B_{j}$, and $C_{k}$ represent the main effects of *B. subtilis*, WP, and *S. cerevisiae*, respectively, ${\left(AB\right)}_{ij}$ is the two-way interaction between *B. subtilis* and WP, ${\left(AC\right)}_{ik}\;$is the two-way interaction between *B. subtilis* and *S. cerevisiae*, ${\left(BC\right)}_{jk}$ is the two-way interaction between WP and *S. cerevisiae*, ${\left(ABC\right)}_{ijk}$ is the three-way interaction effect, and $e_{ijk}$is the random error associated with the observation. Also, the following model was used to compare treatment means when an interaction effect showed to be significant:

(3) $$Y_{ij}=\mu +t_{j}+e_{ij}\;$$

## Results and Discussion

### Egg production, egg weight, and egg mass

Table 2 presents the effects of dietary supplementation with WP, *S. cerevisiae*, and *B. subtilis* on egg production, egg weight, and egg mass during 75–80, 81–86, and 75–86 weeks. A significant interaction effect between WP and *B. subtilis* was observed for egg production in the first period (75–80 weeks, *P* = 0.039) and entire period (75–86 weeks, *P* = 0.069), egg weight (*P* = 0.041), and egg mass in the first period (*P* = 0.009).

**Table 2:** The effect of adding whey powder (WP), *S. cerevisiae* (S. c) and *B. subtilis* (BAS) to the diet of Lohmann laying hens on the parameters of egg production, egg weight and egg mass in 80 - 75, 81-86, 75-86 weeks.

				Egg Production (H.D. %)			Egg Weight (g)			Egg Mass (g)		
Effects	BAS	S. c	WP	75-80 wk	81-86 wk	75-86 wk	75-80 wk	81-86 wk	75-86 wk	75-80 wk	81-86 wk	75-86 wk
Treatment means												
1	-	-	-	84.99	87.24	86.15	59.29	61.89	60.65	50.40	53.98	52.24
2	+	-	-	83.73	82.19	82.87	59.26	60.90	60.09	49.59	50.06	49.78
3	-	+	-	89.62	85.32	88.98	60.45	62.67	61.57	54.15	53.54	54.77
4	-	-	+	78.73	80.93	79.98	58.30	62.54	61.49	45.90	50.63	49.20
5	-	+	+	82.46	83.64	83.06	59.44	62.42	60.98	49.03	52.19	50.66
6	+	-	+	84.52	81.18	82.81	61.07	63.38	62.23	51.62	51.48	51.55
7	+	+	-	85.08	86.48	85.79	60.16	62.74	61.48	51.18	54.26	52.75
8	+	+	+	83.82	82.81	83.41	59.94	62.39	61.19	50.28	51.71	51.08
2-way interaction WP×BAS												
	-		-	87.31^a^	86.28	87.57^a^	59.87^ab^	62.28	61.11	52.27^a^	53.76	53.51^a^
	+		-	84.41^ab^	84.33	84.33^ab^	59.71^ab^	61.82	60.78	50.38^a^	52.16	51.27^ab^
	-		+	80.60 ^b^	82.29	81.52^c^	58.87^b^	62.48	61.23	47.46^b^	51.41	49.93^b^
	+		+	84.17 ^ab^	81.99	83.11^bc^	60.50^a^	62.88	61.71	50.95^a^	51.60	51.31^ab^
2-way interaction WP× *S*. c												
		-	-	84.37	84.71	84.51	59.28	61.40^b^	60.37^b^	49.99	52.02	51.01
		+	-	87.35	85.89	87.39	60.30	62.70^a^	61.52^ab^	52.66	53.90	53.76
		-	+	81.63	81.05	81.39	59.68	62.96^a^	61.86^a^	48.76	51.06	50.37
		+	+	83.14	83.22	83.24	59.69	62.40^ab^	61.08^ab^	49.65	51.95	50.87
SEM				1.522	1.475	1.291	0.426	0.433	0.430	0.980	1.090	0.910
Main effects												
WP			-	85.86^a^	85.30^a^	85.95^a^	59.79	62.05	60.95	51.32^a^	52.96	52.39
			+	82.38^b^	82.14^b^	82.32^b^	59.69	62.68	61.47	49.21^b^	51.50	50.62
S. c		-		83.00	82.88	82.95	59.48	62.18	61.11	49.38	51.53	50.69
		+		85.24	84.56	85.31	60.00	62.55	61.30	51.16	52.93	52.31
BAS	-			83.95	84.28	84.54	59.37	62.38	61.17	49.87	52.59	51.72
	+			84.29	83.16	83.72	60.11	62.35	61.25	50.67	51.88	51.29
SEM				1.077	1.043	0.913	0.301	0.206	0.304	0.693	0.771	0.643
P-Value												
WP				0.028	0.038	0.008	0.813	0.154	0.228	0.036	0.186	0.060
S. c				0.148	0.263	0.075	0.234	0.389	0.664	0.077	0.210	0.082
BAS				0.827	0.453	0.527	0.092	0.954	0.859	0.420	0.519	0.642
WP × *S*. c			0.631	0.738	0.691		0.038	0.031		0.653	0.223	
WP × BAS			0.039	0.577	0.069		0.324	0.359		0.417	0.053	
S. *c* × BAS			0.213	0.389	0.646		0.912	0.969		0.450	0.687	
S. *c* × BAS×WP		0.849	0.225	0.621		0.245	0.565		0.558	0.521		

WP: 1g/kg of whey powder; S. c: 3g/kg *Saccharomyces cerevisiae*(5 × 10^9^CFU); BAS:1g/kg of diet *Bacillus subtilis* (10^9^ CFU).

Means labeled with different superscripts (a, b, c) within the same column for a main effect, two-way interaction, or treatment indicate significant differences at *P* < 0.05.

Sample size (n) = 8 treatments, each with 6 replicates and 6 birds per replicates, with daily sampling performed on all birds throughout the study.

2-or-3-way interaction separated by Duncan.

For egg weight, hens receiving both WP and *B. subtilis* had the highest value (60.50 ± 0.426 g), while those receiving WP without *B. subtilis* had the lowest (58.87 ± 0.426 g, *P* = 0.041). A significant interaction between WP and *B. subtilis* was observed for egg mass in the first period (75–80 weeks, *P* = 0.009). Hens receiving both WP and *B. subtilis* had higher egg mass (50.95 ± 0.980 g) compared to those receiving WP without *B. subtilis* (47.46 ± 0.980 g). The observed interaction between WP and *B. subtilis* on egg weight and egg mass may be attributed to complementary effects on nutrient digestibility and intestinal health. WP is a rich source of high-quality protein and essential amino acids, which are crucial for egg formation and overall productivity (Gangurde et al., 2011). Additionally, WP contains bioactive peptides and water-soluble vitamins that can enhance nutrient absorption (Mann et al., 2019)

*B. subtilis* is a well-known probiotic that improves gut health by modulating the intestinal microflora, enhancing enzyme activity, and increasing villus height, which collectively promote better nutrient utilization (Sen et al., 2012; Zhang and Kim, 2014). When WP and *B. subtilis* are combined, the improved gut environment provided by the probiotic may further facilitate the absorption of nutrients supplied by WP, resulting in greater improvements in egg weight and egg mass than either additive alone.

A significant interaction between WP and *S. cerevisiae* was observed for egg weight during both the second period (*P* = 0.038) and the entire period (*P* = 0.031). In the second period, the highest egg weight was observed in hens receiving WP and *S. cerevisiae* alone (62.96 ± 0.433 and 62.70 ± 0.433 g, respectively), while the lowest was in the group without both supplements (61.40 ± 0.433 g). A similar trend was observed over the entire period, with the highest egg weight in the WP-only group (61.86 ± 0.43 g) and the lowest in the group without both supplements (60.37 ± 0.43 g).

*S. cerevisiae* supplementation in animal diets has been reported to increase the activities of α-amylase, trypsin, and chymotrypsin in the duodenal chyme, which may enhance protein digestibility and nutrient utilization (Ahiwe et al., 2020). Also, yeast is a rich source of nutrients and may act as an efficient substrate for beneficial microorganisms, leading to increased proliferation of gut microbiota (Zhang et al., 2005). These results are consistent with the findings of Hilmi et al. (2020), who stated that protein and amino acid content are important factors in egg weight. Since protein makes up approximately 50 % of egg dry matter, amino acid supplementation is essential during the egg-forming process. The improvement in egg weight in the treatment receiving whey protein could be due to the fact that dried WP provides a balanced amino acid profile (Al-Ubaidi and Bird, 1964), a high protein efficiency ratio (Susmel et al., 1995) and is a rich source of water-soluble vitamins (Modler, 1987). WP can serve as an excellent source of protein supplements. Furthermore, there are some reports showing WP up to 4 percent increases fat and protein digestibility (Balloun and Khajarern, 1974; Susmel et al., 1995) and increases absorption of some minerals like calcium and phosphorous (Al-Ubaidi and Bird, 1964). The three-way interaction among WP, *S. cerevisiae*, and *B. subtilis* was not significant for egg production, egg weight, or egg mass during any period (*P* > 0.05). Since very little research has examined the combined effects of these three factors, there is limited information available in the literature, and further studies are needed.

### Feed intake, feed conversion ratio

The effect of experimental treatments on feed intake and FCR of Lohmann laying hens is summarized in Table 3.

**Table 3:** The effect of adding whey powder (WP), *S. cerevisiae* (S.c) and *B. subtilis* (BAS) to the diet of Lohmann laying hens on feed intake and feed conversion ratio in 75-80, 81-86, 75-86 weeks.

Effects				Feed Intake (g/hen/day)			Feed Conversion Ratio		
	BAS	S. c	WP	75-80 wk	81-86 wk	75-86 wk	75-80 wk	81-86 wk	75-86 wk
1	-	-	-	103.44	105.71	103.44	2.06	1.96	2.01
2	+	-	-	103.11	103.82	103.11	2.10	2.08	2.09
3	-	+	-	104.47	104.42	104.47	1.93	1.97	1.92
4	-	-	+	101.41	104.08	101.42	2.21	2.06	2.09
5	-	+	+	101.38	104.12	101.38	2.07	2.00	2.04
6	+	-	+	102.82	103.40	102.82	2.00	2.01	2.00
7	+	+	-	101.76	103.39	101.76	1.99	1.91	1.95
8	+	+	+	102.82	105.66	102.82	2.05	2.05	2.05
WP			-	103.19	104.34	103.96	2.02	1.98	1.99
			+	102.11	104.23	103.27	2.09	2.03	2.05
S. c		-		102.70	104.17	103.50	2.09	2.03	2.04
		+		102.61	104.40	103.72	2.01	1.98	1.99
BAS	-			102.68	104.58	103.85	2.07	2.00	2.01
	+			102.63	103.99	103.38	2.04	2.01	2.02
SEM				0.861	0.873	0.769	0.458	0.031	0.026
P-Value									
WP				0.378	0.934	0.532	0.144	0.232	0.129
S. c				0.942	0.852	0.843	0.090	0.299	0.111
BAS				0.967	0.631	0.671	0.458	0.740	0.792
WP × *S*. c				0.953	0.384	0.653	0.393	0.432	0.153
WP × BAS				0.234	0.491	0.255	0.054	0.732	0.196
S. *c* × BAS				0.635	0.492	0.944	0.208	0.669	0.695
S. *c* × BAS×WP				0.624	0.735	0.568	0.344	0.102	0.269

Experimental diets had no effects on FI and FCR for the total period (*P* > 0.05). The results of the current study, however, did not support our hypothesis on the synergistic effects of supplementing the diet with *B. subtilis, S. cerevisiae*, and WP. Many studies have demonstrated that the effects of probiotics and prebiotics are influenced by factors such as the species of organisms, the type of host animal, the dosage, the health and nutritional status of the birds, age, physiological stress levels, host genetics, and so forth (Aalaei et al., 2019; Chichlowski et al., 2007; Murawska, 2017). Their effects are more pronounced at younger ages and during peak production periods. In older birds, the physiological response to supplements diminishes, and the ability to enhance performance becomes increasingly limited. Additionally, with advancing age, feed efficiency and feed conversion ratios negatively decrease (Ningsih et al., 2023). It seems that the age of the birds in this study likely masked any positive effects of the prebiotics and probiotics. In research Jazi et al. (2020) on Japanese quails using WP, *B. subtilis* supplements, and a mixture of the two, contrary to our observations, the results showed that the diet containing WP, *B. subtilis*, and MIX improves BWG, and FCR for the whole growing period. The growth-promoting effects of WP on bird performance could be attributed to the lactose fermentation and SCFAs production, which favor beneficial bacteria such as LAB, and improve intestinal integrity. Alloui and Szczurek (2017); Ocejo et al. (2017) displayed that laying hens fed diets containing *S. cerevisiae* at a level of 0.2 % showed the lowest feed consumption. Contrary to these findings, Yalçin et al. (2008) found that except for egg weight, other performance parameters were not significantly affected by diets containing *S. cerevisiae* at levels of 4–20 % in laying quails. Other researchers also reported that FCR does not seem to be affected by probiotics (Fathi et al., 2018; Mohan et al., 1995; Nahashon et al., 1994; Tortuero and Fernandez, 1995). Similarly, other researchers have concluded in several studies that probiotics used as feed additives do not affect egg production traits (Arpášová et al., 2012; Sobczak and Kozłowski, 2015), which is consistent with our research. Taken together, these findings suggest that the lack of significant effects in the present study may be attributed to the advanced age of the hens, as well as potential differences in experimental conditions, dosage, and duration compared to previous research.

### Egg quality

Table 4 showcase the analysis of the egg quality data. A significant interaction effect between WP and *B. subtilis* was observed for yolk height (*P* = 0.017). The highest yolk height was recorded in hens receiving WP without *B. subtilis* (17.53 ± 0.193 mm), while the lowest value was observed in the group receiving both WP and *B. subtilis* (16.88 ± 0.193 mm). These results indicate that the combination of WP and *B. subtilis* did not have an additive effect on yolk height, and their interaction may influence this egg quality parameter in laying hens. The observed interaction may be attributed to the distinct roles of whey protein and *B. subtilis* in nutrient metabolism and yolk formation. While WP provides high-quality protein and essential amino acids (Ha and Zemel, 2003), *B. subtilis* can improve gut health and nutrient absorption (Sen et al., 2012). However, their simultaneous use may alter nutrient partitioning, resulting in a non-additive effect on yolk height. A significant interaction effect between *S. cerevisiae* and *B. subtilis* was observed for both yolk index (*P* = 0.015) and yolk height (*P* = 0.018). The highest yolk index (0.39 ± 0.005) and yolk height (17.55 ± 0.19 mm) were recorded in hens receiving *S. cerevisiae* without *B. subtilis*. The lowest values for both yolk index (0.37 ± 0.005) and yolk height (16.90 ± 0.19 mm) were observed in the group receiving both *S. cerevisiae* and *B. subtilis*. These results indicate that the combination of *S. cerevisiae* and *B. subtilis* did not have an additive effect and may even reduce yolk index and yolk height compared to when each is used alone in aged laying hens at the end of the production phase. The observed interaction between *S. cerevisiae* and *B. subtilis* on yolk height may be attributed to their distinct and potentially competing roles in modulating gut health and nutrient metabolism. *S. cerevisiae* is known to enhance nutrient digestibility and absorption by improving gut microflora balance and stimulating digestive enzyme activity (Zhang and Kim, 2014). *B. subtilis*, as a probiotic, also promotes gut health and nutrient utilization through its effects on intestinal morphology and microbial composition (Sen et al., 2012). The combination of probiotics may disrupt the intestinal environment and nutrient allocation, potentially leading to a reduction in nutrient deposition within the yolk compared to the effects of individual probiotic use. This suggests that while each probiotic can individually improve certain aspects of egg quality, their combined effect may not always be synergistic and should be carefully considered in diet formulation for laying hens. The exact mechanism of this antagonism requires further research. A significant main effect of *S. cerevisiae* supplementation was observed on yolk color, shell percentage, and eggshell thickness (*P* = 0.011, 0.012, and 0.036, respectively). Hens that received *S. cerevisiae* had a higher yolk color score (5.38 ± 0.13) compared to those that did not receive the supplement (4.88 ± 0.13). In contrast, hens without *S. cerevisiae* supplementation showed higher shell percentage (9.69 ± 0.15 %) and greater eggshell thickness (0.408 ± 0.004 mm) compared to the *S. cerevisiae* group (shell percentage: 9.15 ± 0.15 %; eggshell thickness: 0.390 ± 0.004 mm). These results indicate that dietary *S. cerevisiae* supplementation improved yolk color but reduced shell percentage and eggshell thickness in laying hens (Table 4). In addition, hens fed diets containing WP exhibited significantly higher specific gravity (1.83 ± 0.001) compared to those not receiving WP (1.79 ± 0.001; *P* = 0.015).

**Table 4:** The effect of adding whey powder (WP), *S. cerevisiae (S.c)* and *B. subtilis* (BAS*)* to the diet of Lohmann laying hens on egg quality parameters (86 weeks old).

				Egg quality Parameters											
Effects	BAS	S. c	WP	EL (cm)	YH (mm)	AH (mm)	HU	YI	yolk (%)	YC	SHS	SHT (µm)	Alb (%)	shell (%)	SG
Treatment means															
1	-	-	-	5.79	17.11	6.34	77.75	0.37	28.74	4.00	38.17	0.411	61.67	9.60	1.077
2	+	-	-	5.94	17.95	5.66	70.68	0.39	28.20	4.67	45.23	0.402	62.48	9.32	1.078
3	-	+	-	5.96	16.99	5.65	71.79	0.38	27.11	5.33	37.27	0.396	63.79	9.10	1.080
4	-	-	+	5.83	17.75	5.83	81.09	0.39	28.78	5.33	46.07	0.415	61.46	9.76	1.088
5	-	+	+	6.09	17.83	6.37	77.08	0.40	28.07	5.00	34.83	0.372	63.05	8.87	1.080
6	+	-	+	6.03	17.18	5.83	73.33	0.38	27.21	5.00	49.07	0.413	63.06	9.73	1.085
7	+	+	-	6.03	17.34	5.75	72.49	0.37	28.87	5.34	36.67	0.401	61.67	9.45	1.080
8	+	+	+	6.03	17.34	5.74	72.5	0.38	28.88	5.33	36.66	0.401	61.68	4.44	1.080
2-way interaction WP×BAS															
	-		-	5.94	17.12^ab^	5.90	73.87	0.38	27.97	4.92	37.69	0.40	62.60	9.43	1.080
	+		-	6.08	17.42^ab^	5.74	72.92	0.38	29.15	5.42	37.80	0.39	61.59	9.26	1.079
	-		+	5.98	17.53^a^	6.54	78.51	0.39	27.69	5.17	38.04	0.39	63.01	9.30	1.083
	+		+	6.05	16.88^b^	5.91	74.37	0.377	27.73	5	45.15	0.41	62.58	9.7	1.084
2-way interaction BAS ×*S*.c															
	-	-		5.90	17.10^ab^	6.28	76.89	0.38^ab^	27.75	4.83	41.68	0.41	62.50	9.74	1.083
	+	-		6.14	17.40^ab^	5.73	72.52	0.39^a^	28.21	4.92	41.08	0.40	62.14	9.64	1.081
	-	+		6.03	17.55^a^	6.17	75.49	0.39^a^	27.91	5.25	34.05	0.38	63.11	8.98	1.080
	+	+		5.99	16.90^b^	5.91	74.76	0.37^b^	28.66	5.50	41.88	0.40	62.03	9.32	1.082
SEM				0.085	0.193	0.227	1.804	0.005	0.565	0.188	2.44	0.008	0.656	0.207	0.002
Main effects															
WP			-	6.01	17.27	5.82	73.39	0.38	28.56	5.17	37.75	0.397	62.10	9.34	1.079^b^
			+	6.02	17.21	6.23	76.43	0.38	27.71	5.08	41.60	0.401	62.79	9.50	1.083^a^
S. c		-		6.02	17.25	6.01	74.71	0.38	27.99	4.88^b^	41.38	0.408^a^	62.32	9.69^a^	1.082
		+		6.01	17.23	6.04	75.13	0.38	28.28	5.38^a^	37.97	0.390^b^	62.57	9.15^b^	1.081
BAS	-			5.96	17.32	6.22	76.19	0.38	27.83	5.04	37.87	0.398	62.81	9.36	1.081
	+			6.06	17.15	5.82	76.64	0.38	28.44	5.21	41.48	0.400	62.08	9.48	1.081
SEM				0.060	0.136	0.161	1.276	0.003	0.400	0.133	1.727	0.004	0.464	0.146	0.001
P-Value															
WP				0.899	0.746	0.081	0.099	0.403	0.141	0.660	0.123	0.629	0.293	0.466	0.015
S. c				0.907	0.910	0.885	0.818	0.503	0.602	0.011	0.170	0.036	0.712	0.012	0.690
BAS				0.236	0.378	0.087	0.165	0.503	0.292	0.381	0.147	0.787	0.277	0.573	0.894
WP × *S*. c				0.164	0.865	0.964	0.750	1.000	0.134	0.084	0.756	0.629	0.186	0.925	0.507
WP × BAS				0.669	0.017	0.306	0.382	0.098	0.320	0.084	0.159	0.063	0.664	0.181	0.354
S. *c* × BAS				0.111	0.018	0.533	0.320	0.015	0.803	0.660	0.092	0.110	0.586	0.300	0.235
S. *c* × BAS×WP				0.697	0.953	0.629	0.633	0.615	0.327	0.660	0.446	0.964	0.616	0.276	0.894

WP: 1g/kg of whey powder; S. c: 3g/kg *Saccharomyces cerevisiae*(5 × 10^9^CFU); BAS:1g/kg of diet *Bacillus subtilis* (10^9^ CFU); El: egg length; YH: yolk height: AH: albumen height; HU: haugh unit; YI: yolk index; YC: yolk color; SHS: shell strength; SHT: shell thickness; Alb: albumen: SG: specific gravity.

Means labeled with different superscripts (a, b, c) within the same column for a main effect, two-way interaction, or treatment indicate significant differences at *P* < 0.05.

Sample size (n) = 36 eggs per treatments.

2-or-3-way interaction separated by Duncan.

The three-way interaction among WP, *S. cerevisiae*, and *B. subtilis* did not significantly affect any of the parameters investigated in the present study (*P* > 0.05). In alignment with our research, Forte et al. (2016) did not observe any effect of a diet containing *B. subtilis* on egg quality, but among the physical characteristics of eggs, only yolk color according to the Roche scale was affected by the probiotic, which in our experiment, the yolk color was not affected by *B. subtilis.* However, other researchers showed that probiotic supplements containing *B. subtilis* improve eggshell quality by increasing the calcium concentration in the blood serum of laying hens (Panda et al., 2008). According to the findings of Lemos et al. (2014), adding *S. cerevisiae* cell walls at a rate of 0.15 % to the diet of Japanese laying quails increased both shell thickness and percentage. However, our experiment yielded different results, as the use of *S. cerevisiae* led to a significant decrease in both eggshell thickness and percentage. This disparity may be attributed to a variety of factors, such as the dosage and type of *S. cerevisiae* used, the strain of the bird, the breeding environment conditions, and the age of the birds. It is worth noting that our study employed live yeast, which differs from the approach taken by (Lemos et al., 2014).

### Blood biochemical Parameters

Analysis of the levels of glucose, total cholesterol, triglyceride, total protein, albumin, phosphorus, calcium, uric acid, malondialdehyde, and total antioxidant capacity data is shown in Table 5. A significant interaction effect between *S. cerevisiae* and *B. subtilis* was observed for serum total antioxidant capacity, total protein, and triglyceride concentrations in laying hens (*P* = 0.038, 0.031, and 0.038, respectively). The highest values for total antioxidant capacity (1.30 ± 0.08 mmol/L), total protein (7.11 ± 0.28 g/dL), and triglyceride (3125.00 ± 289.58 mg/dL) were observed in hens receiving *B. subtilis* without *S. cerevisiae*. The lowest values for all three serum parameters were found in hens receiving neither *S. cerevisiae* nor *B. subtilis*, as well as in those receiving both additives. These results indicate that *B. subtilis* supplementation alone most strongly enhanced serum antioxidant status, protein, and triglyceride levels, whereas the combination of *S. cerevisiae* and *B. subtilis* did not show an additive effect and, in some cases, resulted in values similar to or lower than those observed in the group receiving neither supplement.

**Table 5:** The effect of adding whey powder (WP), *S. cerevisiae* (S.c) and *B. subtilis* (BAS) to the diet of Lohmann laying hens on blood parameters (86 weeks).

				Blood Parameters									
Effects	BAS	S. c	WP	GLU (mg/dl)	CHO (mg/dl)	TG (mg/dl)	TP (g/dl)	P (mg/dl)	CA (mg/dl)	AL (g/dl)	UA (mg/dl)	MDA (nmol/ml)	TAC (mmol/liter)
Treatment means													
1	-	-	-	221.50	107.31	1545.12	6.28	7.18	32.20	2.48	2.47	2.55^c^	0.92
2	+	-	-	236.25	144.52	2802.50	6.80	7.55	46.63	2.45	3.34	5.65^a^	1.18
3	-	+	-	232.50	117.23	1860.00	6.48	7.95	22.58	2.35	2.22	4.80^ab^	0.98
4	-	-	+	223.51	115.76	1795.00	6.05	6.53	26.18	2.20	2.93	5.83^a^	0.93
5	-	+	+	220.76	107.25	1550.01	6.38	7.40	33.53	2.33	2.38	2.68^c^	0.82
6	+	-	+	243.75	147.21	3447.51	7.43	7.48	37.15	2.38	5.05	5.73^a^	1.42
7	+	+	-	229.00	119.00	1950.10	6.08	4.78	43.60	2.05	3.09	2.03^c^	0.85
8	+	+	+	219.24	113.75	1827.52	6.15	8.15	32.35	2.15	2.49	3.73^bc^	1.00
2-way interaction WP×BAS													
	-		-	227.00	112.25	1702.50	6.38	7.56	27.39^c^	2.41	2.35	3.68	0.950
	+		-	232.63	131.75	2376.25	6.44	6.16	45.11^a^	2.25	3.21	4.44	1.02
	-		+	222.13	111.50	1672.50	6.21	6.96	29.85^bc^	2.26	2.65	4.25	0.88
	+		+	231.50	130.50	2637.50	6.79	7.81	34.75^b^	2.26	3.77	5.30	1.21
SEM				6.554	10.427	289.576	0.276	0.776	2.501	0.092	0.315	0.71	0.078
2-way interaction WP× *S*.c													
		-	-	228.88	125.88	2173.75	6.54	7.36	39.41	2.46	2.91^b^	4.70	1.05
		+	-	230.75	118.13	1905.00	6.28	6.36	33.09	2.20	2.65^b^	3.41	0.917
		-	+	233.63	131.50	2621.25	6.74	7.00	31.66	2.88	3.99^a^	6.35	1.17
		+	+	220.00	110.50	1688.75	6.26	7.78	32.94	2.29	2.43^b^	3.20	0.909
SEM				6.554	10.427	289.580	0.276	0.776	2.500	2.240	0.315	0.710	0.078
2-way interaction BAS ×*S*.c													
	-	-		222.50	111.50	1670.00^b^	6.16^b^	6.85	29.19	2.33	2.70	4.19	0.93^b^
	+	-		240.00	145.88	3125.00^a^	7.11^a^	7.51	41.89	2.41	4.19	6.86	1.30^a^
	-	+		226.63	112.25	1705.00^b^	6.43^ab^	6.68	28.05	2.34	2.29	3.74	0.90^b^
	+	+		224.13	116.38	1888.75^b^	6.11^b^	6.46	37.98	2.10	2.79	2.88	0.93^b^
SEM				6.554	10.430	289.576	0.276	0.776	2.500	0.092	0.314	0.711	0.078
Main effects													
WP			-	229.81	122.00	2039.38	6.41	6.86	36.25	2.33	2.78	3.76	0.98
			+	226.81	121.00	2115.00	6.50	7.38	32.30	2.26	3.10	4.49	1.04
S. c		-		231.25	128.69	2397.50^a^	6.64	7.18	35.54	2.38	3.45^a^	4.94^a^	1.11^a^
		+		225.38	114.31	1796.88^b^	6.27	7.07	33.01	2.22	2.54^b^	3.31^b^	0.91^b^
BAS	-			224.56	111.88	1687.50^b^	6.29	7.26	28.62^b^	2.34	2.50^b^	3.96	0.91^b^
	+			232.06	131.13	2506.88^a^	6.61	6.99	39.93^a^	2.26	3.49^a^	4.29	1.11^a^
SEM				4.635	7.373	204.761	0.195	0.546	1.768	0.065	0.223	0.518	0.055
P-Value													
WP				0.651	0.924	0.693	0.737	0.505	0.127	0.462	0.184	0.339	0.458
S. c				0.379	0.181	0.049	0.194	0.707	0.323	0.102	0.008	0.040	0.017
BAS				0.263	0.077	0.009	0.260	0.726	0.000	0.385	0.004	0.671	0.017
WP × *S*. c				0.249	0.531	0.263	0.704	0.264	0.142	0.259	0.049	0.218	0.395
WP × BAS				0.777	0.981	0.620	0.363	0.160	0.017	0.385	0.696	0.863	0.102
S. *c* × BAS				0.140	0.160	0.038	0.031	0.239	0.584	0.102	0.123	0.128	0.038
S. *c* × BAS×WP				0.895	0.803	0.859	0.547	0.291	0.073	0.840	0.125	0.028	0.837

WP: 1g/kg of whey powder; S. c: 3g/kg *Saccharomyces cerevisiae*(5 × 10^9^CFU); BAS:1g/kg of diet *Bacillus subtilis* (10^9^ CFU); GLU: glucose: CHO: Cholesterol; TG: triglyceride; TP: total protein; AL: albumin; P: phosphorus; CA: calcium; UA: uric acid; MDA: Malondialdehyde; TAC: total antioxidant capacity.

Sample size (n) = = 6 per treatments.

Means labeled with different superscripts (a, b, c) within the same column for a main effect, two-way interaction, or treatment indicate significant differences at *P* < 0.05.

2-or-3-way interaction separated by Duncan.

Agusetyaningsih et al. (2024) found that adding *B. subtilis* to broiler diets raised albumin and total protein levels. *B. subtilis* likely influenced the composition and activity of the intestinal microbiota. A balanced gut microbiota is linked to several physiological functions, such as metabolism and the absorption of nutrients (Abudabos et al., 2020). In addition, probiotics contributed to the production of SCFAs in the intestine of broilers. SCFAs play a role in the absorption of nutrients and may influence protein metabolism (Markowiak-Kopeć and Śliżewska, 2020). Probiotics, including *B. subtilis*, may have indirect effects on liver health, potentially influencing the synthesis of proteins like albumin (Maslennikov et al., 2021). *B. subtilis* is able to form robust biofilms (Vlamakis et al., 2013). Some of researcher proposed that calcium stabilizes biofilms and thereby provides resistance to biofilm dispersion mechanisms (Nishikawa and Kobayashi, 2021). Therefore, this issue can cause more calcium absorption and lead to an increase in blood serum calcium, but further research is needed to confirm this. The experiment found that the three-way interactions of WP, *S. cerevisiae*, and *B. subtilis* in the diet did not have a significant impact on the blood parameters other than malondialdehyde (*P* > 0.05). The reduced interaction effects significantly were detected between WP, *S. cerevisiae*, and *B. subtilis* on the amount of malondialdehyde (*P* < 0.05). The reduction in malondialdehyde levels may be attributed to *S. cerevisiae* components, β-glucan, and yeast mannan oligosaccharides (Matur et al., 2011). Križková et al. (2001) reported that β-glucan and mannan oligosaccharides have antioxidant properties. Also, Yoshida et al. (2009) found that *B. subtilis* can produce carotenoids. ß-carotene is a member of a class of plant pigment molecules referred to as the carotenoids, it acts as provitamin A. It also performs a function as antioxidants.

When *S. cerevisiae* was added to the diet containing WP, compared to when WP was used alone, the blood uric acid level decreased (Table 5; *P* < 0.05). The study demonstrated that incorporating *S. cerevisiae* in the diet alleviated serum uric acid levels, which is consistent with previous findings (Zahira and AL-Tabari, 2014). The reason for this is that *S. cerevisiae* likely activates microorganisms that produce the urease enzyme, which breaks down urea into ammonia in the digestive tract (Yeo and Kim, 1997). Also, adding *S. cerevisiae* to the diet containing *B. subtilis* significantly reduced the amount of triglyceride, total protein, and total antioxidant capacity compared to when *B. subtilis* was used alone. According to the study conducted by Zhang et al. (2022) geese that were fed with yeast experienced a decrease in their triglyceride levels, which aligns with the results of this research. Additionally, Mirza et al. (2020) study that increasing *S. cerevisiae* in the diet of laying hens reduced both blood triglycerides and cholesterol. It is plausible that incorporating live yeast into the diet of broilers could help enhance their fat metabolism (Ahmed et al., 2015). Although our findings suggest that dietary supplementation may increase serum calcium or reduce malondialdehyde levels, these mechanistic explanations remain speculative, as we did not directly measure intermediary parameters such as SCFAs concentrations, gut microbial counts, or gene expression related to calcium absorption and oxidative stress. Therefore, these proposed mechanisms should be considered as hypotheses that require further investigation in future studies.

### Ileum histomorphology

**Fig. 1:**
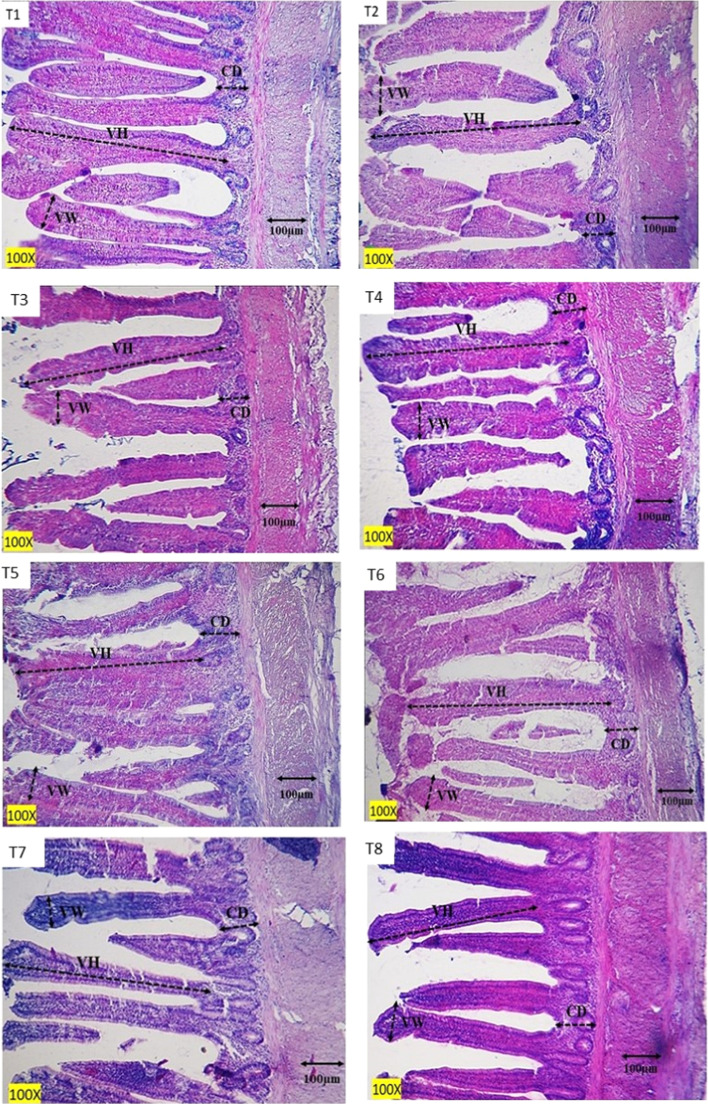
Photomicrographs of ileal tissues from different treatment groups: T1 (control), T2 (*B. subtilis*), T3 (*S. cerevisiae*), T4 (whey powder), T5 (whey powder + *S. cerevisiae*), T6 (*B. subtilis* + whey powder), T7 (*B. subtilis* + *S. cerevisiae*), and T8 (*B. subtilis* + whey powder + *S. cerevisiae*). VH = villus height; VW = villus width; CD = crypt depth. (H&E staining method).

The results of measuring villus height, villus width, and crypt depth of the ileum of Lohmann LSL- lite laying hens are shown in Table 6. Figure 1 show microscopic photography of ileum tissue sections in different treatments. The three-way interaction effects of WP, *S. cerevisiae*, and *B. subtilis* in the diet did not have a significant effect on the histomorphology parameters of the ileum examined in this study (*P* > 0.05). No significant effect was observed between experimental groups in terms of crypt depth and the ratio of villus length to crypt depth in the ileum of laying hens (*P* > 0.05). A significant interaction effect between WP and *B. subtilis* was observed for both villus width (*P* = 0.046) and villus length (*P* = 0.024) in the ileum of aged laying hens at 86 weeks of age. The highest villus width (102.90 ± 1.42 µm) was observed in hens receiving only *B. subtilis*, while the lowest value (97.50 ± 1.42 µm) was found in the group receiving both WP and *B. subtilis*. For villus length, the highest values were recorded in hens receiving only WP (530.23 ± 13.70 µm) as well as only *B. subtilis* (521.15 ± 13.70 µm), whereas the lowest value was observed in the group without both supplements (474.83 ± 13.70 µm).

**Table 6:** The effect of adding whey powder (WP), *S. cerevisiae* (S.c) and *B. subtilis* (BAS) to the diet of Lohmann laying hens on ileum histomorphology parameters (86 weeks).

Effects	BAS	S. c	WP	Histomorphology Parameters			
				VL (µm)	VW (µm)	CD (µm)	VL:CD
Treatment means							
1	-	-	-	479.83	97.40	99.62	4.83
2	+	-	-	509.88	104.39	104.48	4.88
3	-	+	-	469.83	100.65	101.65	4.64
4	-	-	+	526.68	98.51	103.63	5.07
5	-	+	+	533.77	101.03	101.73	5.25
6	+	-	+	521.19	93.76	102.24	5.10
7	+	+	-	532.44	101.42	101.36	5.25
8	+	+	+	495.12	101.23	102.38	4.84
2-way interaction WP×BAS							
	-		-	474.83^b^	99.03^ab^	100.63	4.74
	+		-	521.15^a^	102.90^a^	102.92	5.07
	-		+	530.23^a^	99.77^ab^	102.68	5.16
	+		+	508.15^ab^	97.50^b^	102.30	4.97
SEM				13.698	1.424	1.627	0.143
Main effects							
WP			-	498.00	100.96	101.78	4.90
			+	519.19	98.63	102.50	5.07
S. c		-		509.39	98.53	102.50	4.97
		+		507.79	101.08	101.78	4.99
BAS	-			502.78	99.40	101.65	4.95
	+			514.66	100.20	102.63	5.02
SEM				9.685	1.007	1.150	0.101
P-Value							
WP				0.141	0.121	0.666	0.266
S. c				0.908	0.090	0.667	0.891
BAS				0.389	0.582	0.567	0.644
WP × *S*. c			0.573	0.107	0.919	0.645	
WP × BAS			0.024	0.046	0.425	0.084	
S. *c* × BAS			0.991	0.826	0.639	0.811	
S. *c* × BAS×WP			0.248	0.068	0.285	0.100	

WP: 1g/kg of whey powder; S. c: 3g/kg *Saccharomyces cerevisiae*(5 × 10^9^CFU); BAS:1g/kg of diet *Bacillus subtilis* (10^9^ CFU); VL: villus length; VW: villus width; CD: crypt depth.

Sample size (n) = = 6 per treatments.

Means labeled with different superscripts (a, b) within the same column for a main effect, two-way interaction, or treatment indicate significant differences at *P* < 0.05.

A greater ratio of villus height to crypt depth indicates an improvement in the condition of the intestinal mucosa (Xu et al., 2003). The histological condition of the intestinal mucosa is a good indication of the health status of the bird. So that shorter villi and deeper crypts cause less absorption, increased secretion of mucus into the digestive tract, and cause diarrhea, decreased resistance to disease, and reduced performance (Masouri et al., 2017). The ileum is the primary site for amino acid absorption and longer ileal villi implies higher nutritional utilization reflected in better growth performance (Soren et al., 2024). When the size of the villi is greater it can be regarded as the maturity of the epithelia and such conditions have greater surface area for absorption of nutrients (Roy and Ray, 2023). In this study, no significant effects were found between the experimental groups regarding crypt depth and the ratio of villus length to crypt depth in the ileum of laying hens. In contrast, a previous study by one of the researchers indicated that high amounts of autolysis yeast significantly reduced crypt depth, but did not affect villus height, thickness, or the ratio of villus height to crypt depth in the ileum (Salari and Javidaneh, 2023). The ileum is an essential section for cell membrane transporters and the primary site of amino acid absorption. also longer intestinal villi mean greater utilization of nutrients (Rutz, 2002). In a research conducted in broiler chickens fed with Bacillus, no significant difference was observed in the crypt depth in the duodenum and ileum compared to the control group (Ogbuewu and Mbajiorgu, 2022). Also, in another study, it was found that *B. subtilis* had no significant effect on intestinal morphology in the ileum in pullets (0-6 weeks) (Liu et al., 2020). The crypt is where enterocytes proliferate and differentiate, allowing them to migrate and develop villi (Jayaraman et al., 2017). In an experiment conducted by Samli et al. (2007), no significant effect was observed between the simultaneous use of WP and probiotics, and the use of WP alone regarding villi height. A recent meta-analysis of 25 controlled trials concluded that direct-feeding microbial supplementation was associated with increased small intestinal villus height in broilers (Heak et al., 2017).

WP contains lactose, and lactose is not absorbed in the poultry intestine and is fermented into SCFAs (Gülşen et al., 2002), including lactic acid (Molnár et al., 2018). A review by Markowiak and Śliżewska (2018) detailed how lactic acid produced from bacterial fermentation of sugars is partly dissociated. The undissociated form can pass through lipid cell membranes, where it dissociates and acidifies the cell's contents, inhibiting the growth of pathogenic microorganisms, including putrefactive and Gram-negative bacteria, as well as some molds. Additionally, lactic acid can be fermented into acetic acid, which neutralizes electrochemical cell potential and further decreases the growth of putrefactive bacteria, particularly those from the *Clostridium* and *Salmonella* genera (Markowiak and Śliżewska, 2018). In addition, it is well known that *B. subtilis* produces antimicrobial substances like lipopeptides and bacteriocins (Agusetyaningsih et al., 2024). These compounds may possess antimicrobial properties that aid in preventing the development of harmful bacteria in the gastrointestinal tract. *B. subtilis* may indirectly support intestinal cell integrity by inhibiting the overgrowth of pathogenic microorganisms, maintaining the redox balance and enhancing mucus production that plays a role in intestinal cell protection and integrity (Khan et al., 2023). These changes enhance beneficial bacteria in the intestine, leading to increased growth of gram-positive bacteria and improved intestinal villi.

## Conclusions

In summary, our results showed that, except for malondialdehyde levels, no three-way interactions among WP, *B. subtilis*, and *S. cerevisiae* were observed for the studied parameters in aged laying hens. However, a significant three-way interaction was detected for malondialdehyde, resulting in reduced concentrations. A significant two-way interaction between WP and *B. subtilis* indicated a synergistic effect on egg production, weight, and mass. In contrast, the combined use of *B. subtilis* and *S. cerevisiae* often resulted in antagonistic effects, as co-supplementation reduced yolk index, yolk height, and several serum parameters compared to their individual use. To better elucidate the underlying mechanisms of these additive interactions, further studies assessing SCFA levels, microbial counts, and gene expression in aged laying hens are recommended.

## Animal Ethics

Research Ethics Committees of Kermanshah Razi University has approved the proposal with the approval number of IR.RAZI.REC.1402.070

## Conflict of interest

We wish to confirm that there are no known conflicts of interest associated with this publication and there has been no significant financial support for this work that could have influenced its outcome.

## Funding

No external or direct financial support was received for this study.

## Intellectual Property

We confirm that we have given due consideration to the protection of intellectual property associated with this work and that there are no impediments to publication, including the timing of publication, with respect to intellectual property. In so doing we confirm that we have followed the regulations of our institutions concerning intellectual property.

## Disclosures

The authors declare that they have no known competing financial interests or personal relationships that could have appeared to influence the work reported in this paper.

## Data Availability

Data will be made available on request.
